# Clinical significance of measuring the lateral atlantodental interval in children with tic disorders

**DOI:** 10.3389/fneur.2025.1578149

**Published:** 2025-11-06

**Authors:** Beiru Peng, Runxin Peng, Xiumei Chen, Xuanrui Lu, Chuyu Huang, Lijiao Zhang, Gaofeng Liang

**Affiliations:** 1 The Second Affiliated Hospital of Guangzhou University of Chinese Medicine, Guangdong Provincial Hospital of Chinese Medicine, Guangzhou, China; 2 The Second Clinical College of Guangzhou University of Chinese Medicine, Guangzhou, China; 3 Luo Xiao-Rong Famous Doctor Studio of Guangdong Provincial Hospital of Traditional Chinese Medicine, Guangzhou, China; 4 Guangdong Provincial Second Hospital of Traditional Chinese Medicine, Guangzhou, China

**Keywords:** tic disorders, atlantoaxial joint, craniovertebral junction, lateral atlantodental interval, asymmetry, radiology

## Abstract

**Background:**

The pathophysiology and causation of tic disorders (TD) remain unclear. Clinically, children with TD with head and neck tics, trunk tics, often showing associated spinal abnormalities, may be closely related to the atlantoaxial joint. The purpose of this research is to assess the level of lateral atlantodental interval (LADI) in children with TD and to explore the correlation between TD and the asymmetry of bilateral LADI.

**Methods:**

The variance in the bilateral lateral atlantodental interval (VBLADI) level was investigated in a retrospective analysis of 80 children with TD between July 2021 and December 2024. Meanwhile, examining the correlation between tic symptoms and VBLADI levels based on VBLADI ≤ 2 mm and >2 mm for grouping.

**Results:**

The mean VBLADI among children with TD was 1.32 mm (range: 0.62–2.19 mm). A robust and statistically significant association was identified between head shaking and VBLADI >2.2 mm, even after adjusting for potential confounders and correcting for multiple comparisons [adjusted odds ratio (aOR) = 12.64; FDR *q*-value = 0.035). However, no linear correlation was observed between VBLADI levels and the total Yale Global Tic Severity Scale score (*p* = 0.780).

**Conclusions:**

Asymmetry in the bilateral LADI is associated with TD symptoms, particularly head shaking. Nonetheless, VBLADI levels do not correlate with the overall severity of TD.

## 1 Introduction

Tic disorders (TD), characterized by the presence of motor or vocal tics, are a group of neurodevelopmental disorders with sudden, rapid, recurrent, arrhythmic, stereotyped movements, or vocalizations as the primary clinical manifestation. According to the description in the Diagnostic and Statistical Manual of Mental Disorders-Fifth Edition-Text Revision (DSM-5-TR), TD is divided into the following groups: Tourette's syndrome (TS), chronic motor or vocal tic disorder (CTD), provisional tic disorder (PTD), other specified and unspecified tic disorders (1). In this paper, the overarching term “TD” is used to refer to all primary tic disorders including the categories mentioned above. The symptoms usually decrease in severity later as age progresses. The TD incidence rate was 2.46% in Chinese school students aged 6–16 (2). TD is most common prior to puberty, between 4 and 6 years of age, and severity is mostly in the age range of 10–12 years (3, 4). Numerous environmental, neurological, and genetic variables can be implicated in the causes of TD, however the exact etiology and pathophysiology remain unclear (5).

The most common initial motor tics affect the head and face, with eye, mouth, and nose movements being the most common (6). As the condition progresses, some tics may spread to axial muscle groups such as the trunk, neck, and shoulders (7). The severity varies greatly. Extremely repetitive tics with violent head and neck motion can even lead to abnormalities in the function and structure of the cervical spine (8).

Over the past two decades of research, it has been shown that abnormal cervical spine function, particularly instability of the atlanto-occipital and atlanto-axial joints, may be associated with the occurrence of TD, but the related neurobiological mechanisms still need to be further explored (9). The craniovertebral junction (CVJ), also known as the occipital-atlanto-axial joints, is the most complex joints of the axial skeleton (10). It is composed of the occiput (C0), the atlas (C1), and the axis (C2). C1 is ring-shaped and has no vertebral body, while C2 has a prominent vertebral body called the odontoid process or dens, serving as the pivot point for the 1′s rotation and restricting its translational movement (11–13). Furthermore, the absence of intervertebral discs allows for increased movement and rotation in the joint, thereby favoring mobility at the expense of stability (14).

The atlanto-occipital (C0-C1) and atlantoaxial (C1-C2) joints differ markedly in their function. The C0–1 complex mainly responsible for flexion/extension and lateral flexion, accounting for 50% of the total flexion/extension movements of the neck, but its skeletal structure limits the rotational function (13, 15); while the C1–2 complex is the main joint for cervical rotation, bearing approximately 50% of the total rotational amplitude, with the maximum unilateral rotation reaching 40°, and the range of motion being the widest among all cervical vertebrae (12, 13, 15). The mechanical stability of the CVJ is mainly maintained by ligament structures such as the transverse and the alar ligaments (16). The transverse ligament has the highest strength, while the alar ligament mainly limits lateral flexion (16–18). In addition, the tectorial membrane, capsular ligaments and the neck muscles also provide additional support for the CVJ (19).

The CVJ in children demonstrates unique anatomical and biomechanical characteristics. During development, the pediatric cervical spine undergoes significant structural changes, particularly between ages 3 and 7, with rapid growth of the atlas and axis. The odontoid process fuses with the vertebral body between 4 and 7 years of age, while its apical ossification center fuses around age 12 (20, 21). However, children possess relatively shallow atlantoaxial articular surfaces, highly elastic and lax joint capsules and ligaments, and underdeveloped neck muscles (22). These factors collectively contribute to decreased stability of the CVJ, resulting in a higher incidence of CVJ dysfunction in the pediatric population.

CVJ instability is defined as a pathological change in the anatomical relationship between the articular components, usually caused by insufficiency or rupture of the surrounding stabilizing structures. Damage to ligamentous insertions can cause significant instability, even in the absence of bone fractures (19, 23). Potential triggers include muscle overuse or disuse, physical trauma, and chemical or genetic factors that cause abnormal ligament laxity—all of which may contribute to an increased range of motion in the cervical spine (24, 25). The significance of CVJ instability lies in its potential to compress neural structures, causing nerve damage, and in severe cases, even leading to myelopathy, paralysis, or death (23). There have been previous case reports of children with TD who were also diagnosed with high cervical spinal cord type cervical spondylosis (26, 27).

Clinically, we have observed that children with TD exhibiting motor tics involving the head, neck, and trunk often present with a constellation of spinal abnormalities. Physical examination frequently reveals postural misalignment and muscular tenderness in the cervical and scapular regions, while imaging examinations often show the asymmetry of bilateral LADI, as well as loss of cervical lordosis, etc.

The LADI is the shortest distance from the lateral margin of the odontoid process of the axis to the medial margin of the lateral mass of the atlas (28). The difference between the distances on both sides is VBLADI. A comprehensive study examining VBLADI measurements in 3,072 asymptomatic children was 0.09 ± 1.23 mm (range −6.05 to 4.87 mm) (29). Although studies have long shown that LADIs should be symmetrical in the neutral position (28). Widened LADIs do not necessarily indicate an abnormal atlantoaxial joint, since this could be an anatomical variation that is common in both children and adults (29, 30), or alternatively, signal underlying injury to the cervical ligaments. In conjunction with a study conducted in China, it was defined in this research that the VALADI in children should not surpass 2.2 mm (28, 29, 31).

However, there is a lack of studies specifically investigating the correlation between CVJ instability and tic disorders; however, clinical observations have consistently noted their frequent co-occurrence. This might suggest a connection between tic disorders and CVJ instability.

Thus, this study intends to take the LADI as the entry point. By analyzing the VBLADI values and bilateral asymmetry in the TD population and their correlations with tic symptoms, it aims to further reveal the potential mechanism between TD, VBLADI, and CVJ functions.

## 2 Materials and methods

### 2.1 Study design and population

This study employed a real-world, retrospective case-control design. Data were extracted from the medical records of pediatric patients diagnosed with TD at the pediatric outpatient clinic of Fangcun Hospital, Guangdong Provincial Hospital of Traditional Chinese Medicine. The database covered the period from July 2021 to December 2024. All cases that met the inclusion and exclusion criteria were included in the study. In total, 80 children were included. Ethical approval for this study was obtained from the ethics committee of Guangdong Provincial Hospital of Chinese Medicine. The inclusion criteria were as follows:

(A) Diagnosis of TD based on the Diagnostic and Statistical Manual of Mental Disorders, Fifth Edition, Text Revision (DSM-5-TR) (1);
(B) Age between 4 and 14 years;
(C) Availability of cervical spine X-ray images, including anteroposterior open-mouth atlantoaxial view, and anteroposterior and lateral views.
(D) The time interval between YGTSS assessment and imaging scan was ≤ 7 days.
The exclusion criteria were as follows:

(A) Comorbid neuropsychiatric conditions such as obsessive-compulsive disorder, attention-deficit/hyperactivity disorder, or autism spectrum disorder;
(B) History of spinal trauma, infectious diseases affecting the spine, or prior spinal surgery;
(C) Evidence of cervical deformity on digital radiography (DR).
Based on the VBLADI measurements, subjects were categorized into two groups: the abnormal group (VBLADI >2 mm; *n* = 20) and the normal group (VBLADI ≤ 2 mm; *n* = 60). The primary objective was to compare the clinical characteristics of TD in the two groups.

### 2.2 Data collection

Data were systematically extracted from the electronic medical record (EMR) system using a predefined data collection form covering outpatient diagnoses, demographic information, clinical features of TD, Yale Global Tic Severity Scale (YGTSS) scores, and imaging data. The diagnosis of TD was strictly based on the criteria outlined in the DSM-5-TR. All diagnoses were confirmed by experienced pediatric neurologists in our outpatient clinic. The diagnostic process involved: (1) a detailed clinical interview and neurological examination of the child; (2) a semi-structured interview with the primary caregiver(s) to ascertain the symptom presentation, frequency, intensity, and disease course; and (3) a consensus diagnosis reached by the expert team after reviewing all available clinical information. Only cases with unequivocal diagnoses were included in this study.

All personally identifiable information was removed to ensure participant anonymity. Two independent researchers (Runxin Peng and Xiumei Chen) verified the accuracy and completeness of the extracted data. Any discrepancies were resolved through discussion or by arbitration from a third senior researcher (Beiru Peng).

The YGTSS is a semi-structured interview used to measure the severity of tics, with motor and vocal tics that occurred during the previous week being rated separately on a 0–5 point scale across five dimensions: number, frequency, intensity, complexity, and interference (32). The YGTSS scores used in this study were retrospectively collected from medical records, reflecting initial assessments conducted by trained pediatricians as part of standard clinical practice.

Notably, all imaging examinations were performed after the YGTSS evaluations had been completed. As a result, the researchers responsible for extracting and recording the YGTSS scores were blinded to the participants' group assignments, which were based on subsequent VBLADI measurements.

### 2.3 Imaging measurement

Utilizing the Siemens Ysio Max Digital Radiography System, the atlantoaxial open-mouth photography was carried out as follows: the patient stood, both upper limbs were positioned at the side, and the head was in the median position, vertical to the table and facing the midline. A neutral head position was maintained while the occipital protuberance was positioned 2 cm above the IP board's center. When shooting, the dentate process was perpendicular to the scanning line, the two outer canthus lines were parallel to the scanning plane, and the youngster was asked to open his mouth and make as much of a “ah” sound as he could. This helped to make the shot clearer.

**Figure 1:**
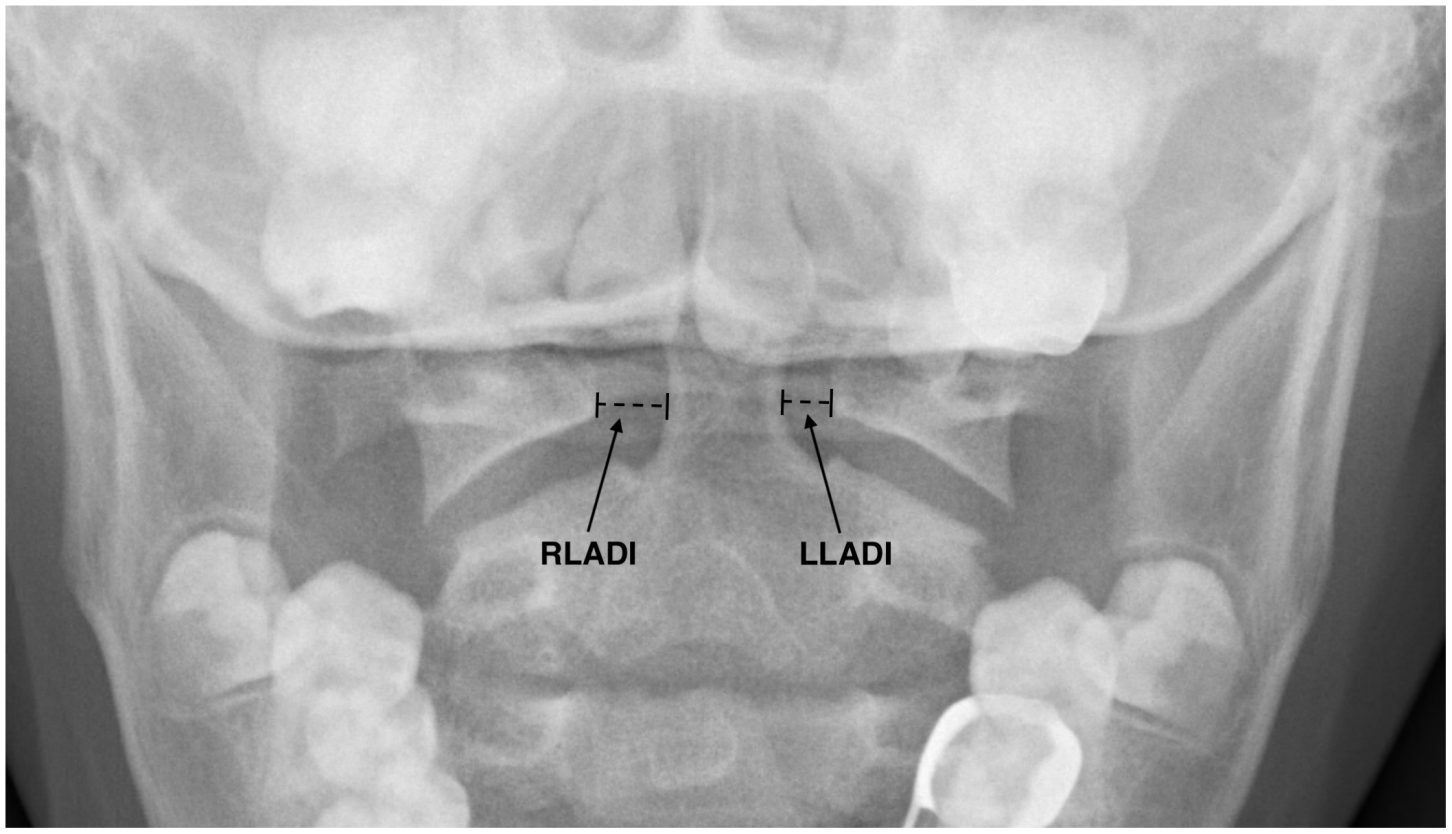
Measurement of the LADI on digital radiography. The dotted lines indicate the shortest distance from the lateral margin of the odontoid process (axis) to the medial margin of the lateral mass (atlas).

This procedure was performed by professional imaging doctors to rule out errors caused by manual measurements, changes in measurement posture, and apparent spinal deformities. The standard tablet showed that the atlas and axial vertebrae were displayed between the upper and lower teeth. The crown of the upper middle incisor overlapped with the base of the occipital bone, while the tooth process of the second cervical vertebra didn't overlap with the bone. The space between the dental process and the first cervical vertebra was symmetrical. The atlas and occipital joints were tangentially displayed, and the texture of the atlas and axial vertebrae were clearly displayed (Figure 1).

We acquired the original images of the atlantoaxial open-mouth radiographs from hospital record database and performed measurements of bilateral LADI using a software tool, Bee Dicom Viewer (an image visualization and measurement system). The difference between the left LADI (LLADI) and right LADI (RLADI) was defined as VBLADI, which was expressed as an absolute value. VBLADI reflected the magnitude of the asymmetry in the gap between the odontoid process of the axis and the lateral process of the atlas, but did not represent the direction. VBLADI was calculated as |RLADI—LLADI|. VBLADI of ≤ 2.2 mm was considered normal values in Chinese children, and a VBLADI of >2.2 mm was considered abnormal (28). Initially, it was an experienced radiologist who was responsible for measuring the lateral atlanto-occipital diameter (VBLADI). This radiologist was unaware of all the clinical data (including the YGTSS scores and the final research hypothesis). After the data collection was completed, all measurements were performed by one author (Xuanrui Lu) and were verified by two other authors (Beiru Peng and Runxin Peng) to eliminate the possibility of interobserver variability.

### 2.4 Statistical analysis

All data were analyzed using SPSS 26.0. LLADI and RLADI values of children with TD that conformed to a normal distribution represented by the mean (standard deviation, SD). Continuous variables of age, YGTSS scores, duration of TD and VBLADI values of children with TD were expressed as median (interquartile range, IQR). The remaining categorical variables were expressed as *n* (%). The chi-square test was used for intergroup comparisons of classified variables, and Fisher's exact test when conditions were not met. Two or more groups of continuous variables conforming to normal distribution were compared by analysis of variance (ANOVA). Data that were not normally distributed were compared using rank sum tests such as Kruskal–Wallis test. Correlation analysis between YGTSS scores and VBLADI values was used with Spearman's rank correlation. Multivariate logistic regression was used to detect the association between the incidence of TD symptoms and >2.2 mm or ≤ 2.2 mm of VBLADI levels.

Given that multiple statistical comparisons were performed across various tic symptoms, we applied False Discovery Rate (FDR) correction to control the risk of Type I errors (false positives). The Benjamini–Hochberg (B–H) procedure was applied to the raw *p*-values from all comparisons in the multivariate logistic regression model (Model 2), with an FDR threshold set at 5% (*Q* = 0.05). The adjusted *p*-values are reported as FDR *q*-values. An association was considered statistically significant only if the *q*-value was less than 0.05.

As a real-world retrospective study, the sample size was determined by the total number of eligible patients during the specified study period (*n* = 80). To assess the statistical power for the primary findings, a *post-hoc* power analysis was conducted after data analysis. This analysis was performed specifically for the primary outcomes that remained statistically significant after FDR correction. Using G^*^Power software (version 3.1.9.2), the effect size (*f*^2^) was calculated based on the adjusted Odds Ratio (aOR) observed in the multivariate logistic regression model (Model 2). The analysis parameters included: α = 0.05, total sample size *N* = 80, and was adjusted for the total number of predictors in the model. A statistical power of >80% was considered sufficient.

All comparisons used 2-sided tests at a 0.05 level of significance. The null hypothesis for all analyses was that there was no difference between the study groups.

## 3 Results

### 3.1 General information about the subjects

This study included 80 children with TD who met the inclusion and exclusion criteria, of which 64 were male (80.0%) and 16 were female (20.0%). The age range was from 4.5 to 13 years, with a median and quartile of 9 (7, 10) years. Among them, there were 37 cases of PTD (46.3%), 30 cases of CTD (37.5%), and 13 cases of TS (16.3%). The median and quartiles of TD duration and YGTSS scores were 0.5 (0.125, 2.0) years and 20 (17, 23.75).

### 3.2 LADI levels in children with TD

The mean value and standard deviation of LLADI and RLADI in children with TD are 5.03 ± 1.52 mm and 5.47 ± 1.43 mm, and the median and quartile of VBLADI are 1.32 (0.62, 2.19) mm. In PTD, CTD and TS groups, there is no significant difference between LLADI, RLADI and VBLADI (Table 1).

**Table 1:** Comparison of LLADI, RLADI, and VBLADI in different types of TD.

**Types**	**Total**	**PTD**	**CTD**	**TS**	***p*-value**
LLADI (mm)	5.03 ± 1.52	5.21 ± 1.64	4.87 ± 1.37	4.90 ± 1.55	0.269
RLADI (mm)	5.47 ± 1.43	5.03 ± 1.52	5.34 ± 1.37	5.76 ± 1.47	0.226
VBLADI (mm)	1.32 (0.62, 2.19)	1.30 (0.33, 2.30)	1.21 (1.21, 1.94)	1.94 (0.87, 2.79)	0.364

PTD, provisional tic disorder; CTD, chronic motor or vocal tic disorder; TS, Tourette's syndrome.

### 3.3 Relationships of VBLADI and YGTSS scores

The association coefficients of VBLADI and YGTSS scores in children with TD are provided in Table 2. No obvious correlation is observed between VBLADI level and YGTSS total score (*p* > 0.05).

**Table 2:** Correlation between VBLADI and YGTSS scores in children with TD.

**Types**	**YGTSS scores**	**VBLADI (mm)**	***r***	***p*-value**
*N* = 80	20 (17, 23.75)	1.32 (0.62, 2.19)	0.034	0.780

YGTSS, The Yale Global Tic Severity Scale.

### 3.4 Relationships of VBLADI and clinical symptoms of TD

After organizing the medical data, it is concluded that the symptoms of TD exhibited by the children fall into the following four major categories: facial tics, head and shoulder tics, trunk and limb tics, and voice tics. Among them, facial tics include eye movements, nasal movements, and mouth movements. Head and shoulder tics include shaking head, swiveling head, nodding or raising head, stretching or shrinking neck, and shrugging shoulder. Trunk and limb tics include upper limb, lower limb, abdominal muscles, and trunk movements. In total, there are 13 tic symptoms (Table 3).

**Table 3:** Comparison of clinical features in children with TD between normal group and abnormal group.

**Types**	**Normal group (*n* = 60)**	**Abnormal group (*n* = 20)**	**χ^2^**	***p*-value**
**Clinical symptoms**				
**Facial tics**				
Eye movements	39 (65.0%)	11 (55.0%)	0.640	0.424
Mouth movements	23 (38.3%)	11 (55.0%)	1.705	0.192
Nasal movements	13 (21.7%)	8 (40.0%)	2.604	0.107
**Head and shoulder tics**				
Shaking head	10 (16.7%)	9 (45.0%)		0.015^a, b^
Swiveling head	31 (51.7%)	6 (30.0%)	2.833	0.092
Nodding or raising head	17 (28.3%)	7 (35.0%)	0.317	0.573
Stretching or shrinking neck	5 (8.3%)	4 (20.0%)		0.217^b^
Shrugging shoulder	22 (36.7%)	5 (25.0%)	0.913	0.339
**Trunk and limbs tics**				
Upper limb	10 (16.7%)	5 (25.0%)		0.509^b^
Lower limb	5 (8.3%)	1 (5.0%)		1.000^b^
Abdominal muscles	4 (6.7%)	3 (15.0%)		0.358^b^
Trunk movements	2 (3.3%)	4 (20.0%)		0.032^a, b^
Voice tics	15 (25.0%)	10 (50.0%)	4.364	0.037^a^
**Clinical signs**				
Shoulder and neck tenderness	29 (48.3%)	7 (35.0%)	1.077	0.299

Normal group: VBLADI ≤ 2.2 mm; Abnormal group: VBLADI >2.2 mm.

^a^*p*-value < 0.05.

^b^Fisher's exact test.

**Figure 2:**
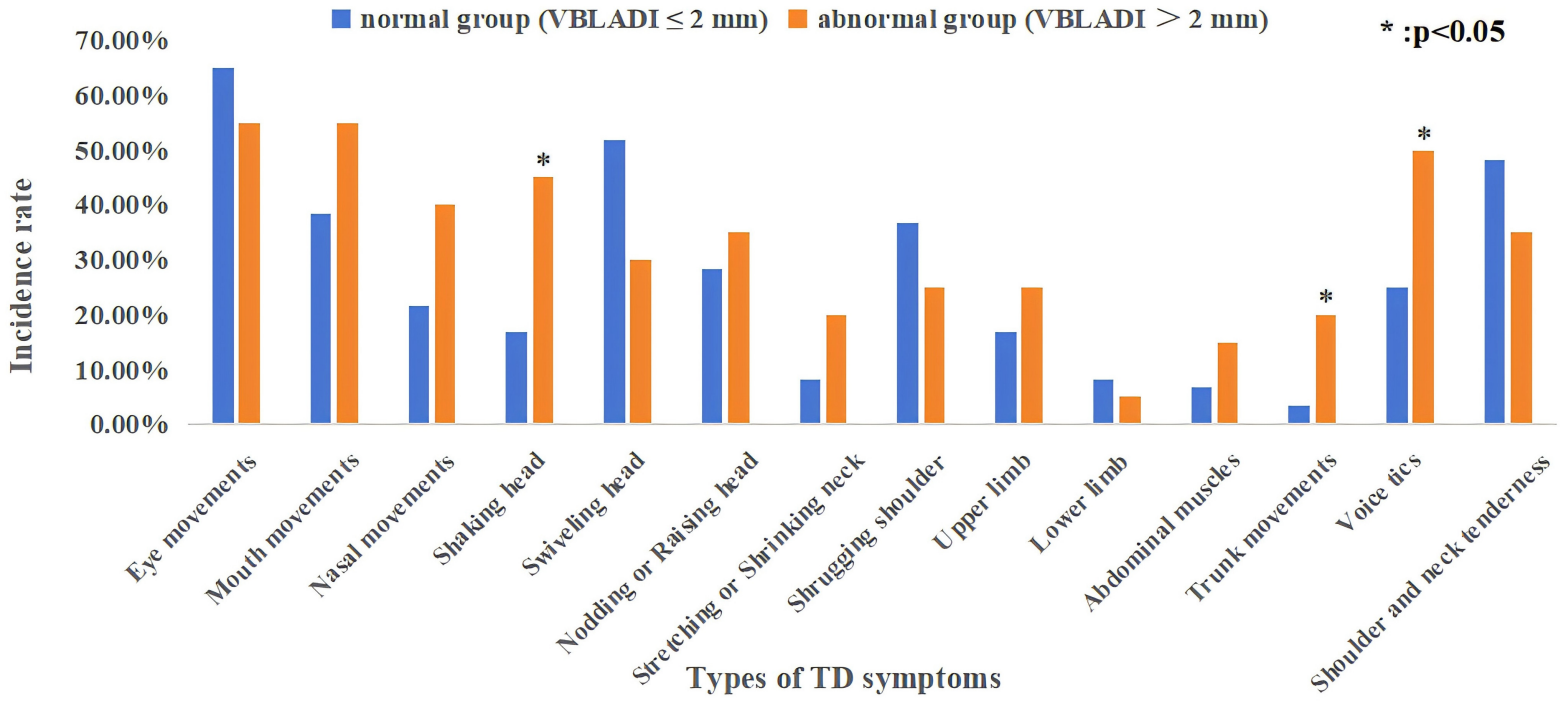
The incidence distribution of TD symptoms in the normal group and the abnormal group. p-value < 0.05 means: Taking the symptom of “Shaking head” as an example, the incidence rate in the abnormal group is higher than that in the normal group, and this difference is statistically significant.

In this study, 80 children with TD were divided into a normal group and an abnormal group based on VBLADI ≤ 2.2 mm and VBLADI >2.2 mm. Of which, there are 60 cases (75.0%) in the normal group and 20 cases (25.0%) in the abnormal group. Table 3 describes the incidence of clinical characteristics of TD between the two groups. In these three clinical symptoms of shaking head, trunk movements, and vocal tics, the incidence of the abnormal group is higher than the normal group, with statistically significant differences (*p* = 0.015, *p* = 0.032, *p* = 0.037). While in the remaining clinical symptoms of TD, including the facial tic movements, swiveling head, nodding or raising head, stretching or shrinking neck, shrugging shoulder, upper limb tics, lower limb tics, abdominal muscles tics, and shoulder and neck tenderness, there is no statistical significance found for the incidence rates in both groups (*p* > 0.05). The distribution of TD symptoms incidence is shown in Figure 2.

Table 4 shows the odds ratio (ORs) and 95% confidence intervals (CIs) for VBLADI level to tic symptoms. Based on the results of the chi-square analysis in Table 3, independent variables with *p*-values less than 0.05 are included as the accession model, including shaking head, trunk movements and voice tics. Combined with clinical observations, head and neck symptoms may be associated with VBLADI level, so symptoms of swiveling head, nodding or raising head, stretching or shrinking neck, and shrugging shoulder were also included in this model.

**Table 4:** Logistic regression models showing association between VBLADI level and tic symptoms.

**Variable**	**Group**	**Model 1**			**Model 2**		
		β	**OR (95% CI)**	***p*** **-value**	β	**OR (95% CI)**	***p*** **-value**
Shaking head	1						
	2	1.54	4.666 (1.164–18.710)	0.030^a^	2.357	12.639 (2.045–78.119)	0.006^a^
Swiveling head	1						
	2	0.147	1.158 (0.289–4.646)	0.836	0.147	1.158 (0.254–5.284)	0.850
Nodding or raising head	1						
	2	0.858	2.359 (0.645–8.627)	0.194	0.987	2.684 (0.645–11.167)	0.175
Stretching or shrinking neck	1						
	2	1.859	6.419 (1.170–35.223)	0.032^a^	2.196	8.992 (1.163–69.535)	0.035^a^
Shrugging shoulder	1						
	2	−0.693	0.500 (0.123–2.023)	0.331	−0.434	0.648 (0.142–2.961)	0.576
Trunking shake	1						
	2	1.985	7.282 (0.806–65.798)	0.077	2.503	12.222 (1.180–126.566)	0.036^a^
Voice tics	1						
	2	0.695	2.004 (0.566–7.098)	0.281	0.096	1.1 (0.227–5.328)	0.905

Group 1: Normal group (VBLADI level ≤ 2 mm); Group 2: Abnormal group (VBLADI level >2 mm); Model 1: unadjusted; Model 2: adjusting mixed factors of age, sex, duration, types of TD, and YGTSS scores.

^a^*p*-value < 0.05.

OR, odds ratio; CI, confidence interval.

Finally, the resulting logistic model is statistically significant, with χ^2^ being 27.387 and *p* being 0.011. The model is able to correctly classify 82.5% of the study subjects. The sensitivity of the model is 55.0%, and the specificity is 97.7%. Multivariate logistic regression analysis reveals that, in adjusting for age, sex, duration, types of TD, and YGTSS scores, after that, the incidence of shaking head (adjusted OR 12.639; 95% confidence interval, 2.045–78.119; *p* = 0.006), stretching or shrinking neck (8.992; 1.163–69.535; 0.035), and trunk movements (12.222; 1.180–126.566; 0.036) show a significant correlation with VBLADI >2 mm. In general, larger OR suggests higher risk factors. These three symptoms are the risk factors for the asymmetry of the bilateral atlantoaxial interval. The results are shown in Table 4.

To control the increased risk of false positives due to multiple testing, the FDR was controlled at 5% using the B–H procedure for all seven comparisons. After FDR adjustment, only the association for shaking head remained statistically significant (*q* = 0.035). Although the raw *p*-values for trunking shake (*p* = 0.036) and stretching or shrinking neck (*p* = 0.035) were below 0.05, their FDR *q*-values were 0.050, failing to meet the pre-specified significance threshold.

A *post-hoc* power analysis indicated that for the primary finding of shaking head (aOR = 12.639), our sample size of 80 provided exceedingly high statistical power (Power >99.9%, α = 0.05). This confirms that the study was amply powered to detect this strong and clinically relevant association. The results are shown in Table 5.

**Table 5:** Association between various tic symptoms and VBLADI >2.2 mm in multivariable logistic regression model (Model 2) with FDR correction.

**Variable**	**β (Model 2)**	**OR (95% CI) (Model 2)**	***p*-value (Model 2)**	**FDR *q*-value**
Shaking head	2.357	12.639 (2.045–78.119)	0.006	0.035^a^
Trunking shake	2.503	12.222 (1.180–126.566)	0.036	0.050
Stretching or shrinking neck	2.196	8.992 (1.163–69.535)	0.035	0.050
Nodding or raising head	0.987	2.684 (0.645–11.167)	0.175	0.050
Shrugging shoulder	−0.434	0.648 (0.142–2.961)	0.576	0.050
Swiveling head	0.147	1.158 (0.254–5.284)	0.850	0.050
Voice tics	0.096	1.100 (0.227–5.328)	0.905	0.050

^a^*q*-value < 0.05.

FDR, false discovery rate.

## 4 Discussion

Compared with the extensive literature on the biomechanics of the adult cervical spine, there is a relative paucity of research on the pediatric cervical spine (33). This is particularly evident in the context of neurodevelopmental disorders such as TD, where studies investigating their association with cervical biomechanics in children are even scarcer. Previous investigations have primarily been documented as case reports, focusing on clinical features of TD patients with notable structural cervical injuries—including conditions such as cervical spondylotic myelopathy, fractures, and atlantoaxial subluxation (34–36). However, no studies have yet reported on the relationship between TD and atlantoaxial functional impairments, such as CVJ instability.

To address this research gap, we conducted the present study, which yielded three principal findings:

First, the mean VBLADI value among children with TD was established as 1.32 (0.62, 2.19) mm.

Second, a correlation was observed between VBLADI and the presence of TD symptoms—particularly head shaking.

Third, no direct correlation was observed between overall TD severity and the degree of VBLADI.

Consistent with the first finding, our results indicated that the mean VBLADI in children with TD is comparable to values reported in typically developing children (29). This suggests that a certain degree of VBLADI asymmetry may be a common anatomical variant in the pediatric population and is not specific to TD. Previous studies have indicated that LADI asymmetry may be associated with CVJ instability. Potential causes include ligamentous laxity, trauma, syndromic, iatrogenic, congenital and inflammatory (37). It is more likely to occur in children, and the mechanism may be related to the physiological characteristics of the atlantoid structure in children. In childhood, immature osseous structures, increased ligamentous laxity, and insufficient muscle strength lead to atlantoaxial instability (22). Immature spinal ligaments are relatively lax, the facet joints are shallow and horizontally oriented, and the physiological anterior wedging of the undeveloped spinous processes and vertebral bodies contribute to high torque and shear forces acting on the pediatric cervical spine, resulting in greater mobility (22). In addition, due to a larger head-to-neck ratio compared to adults, poor neck balance, and weaker muscular and ligamentous support, children are highly susceptible to injuries to both the head and cervical spine (38).

**Figure 3:**
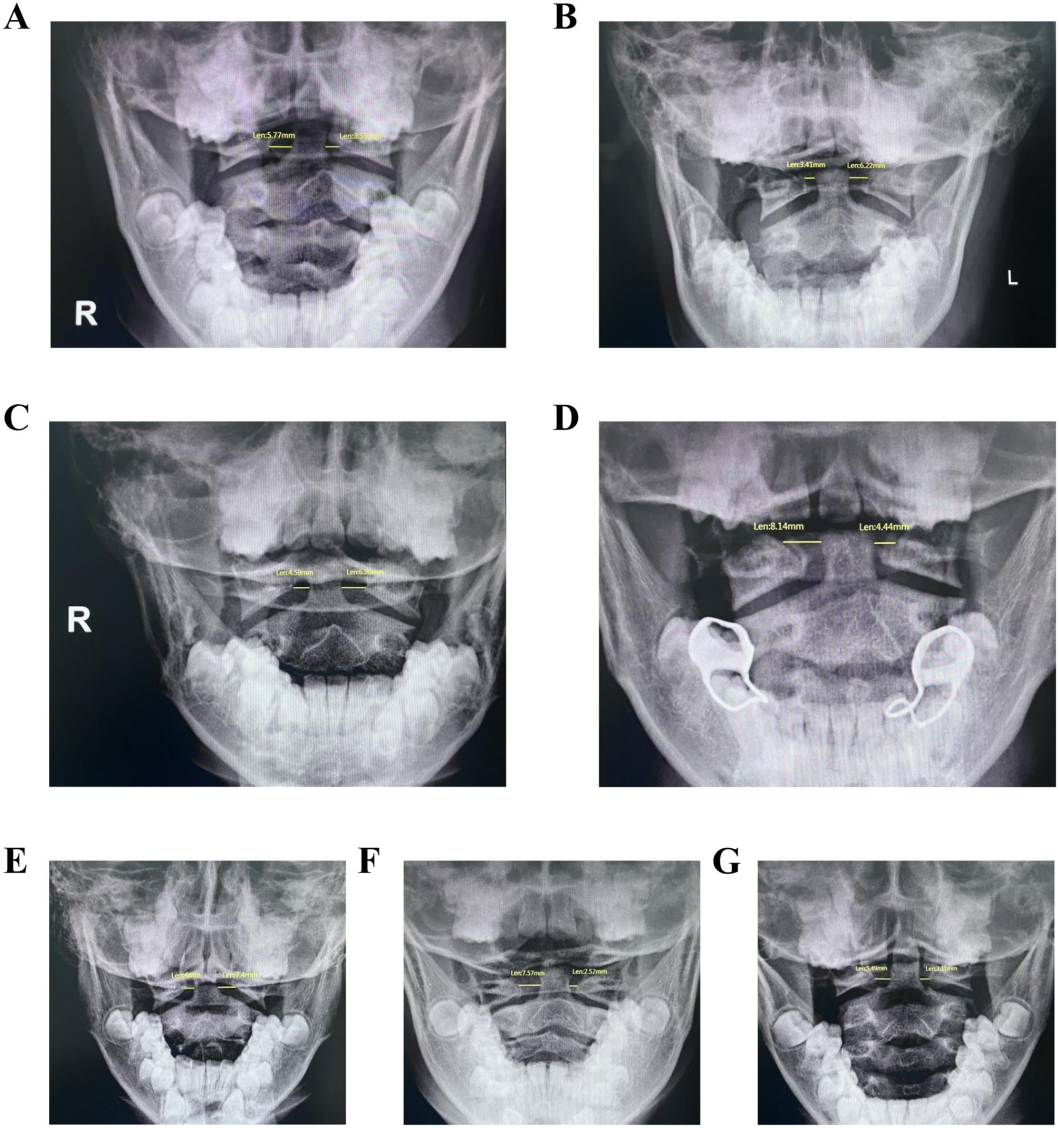
**(A–G)** The X-ray manifestations of the atlantoaxial opening position in children with TD of different ages and genders. The left side of the image corresponds to the right side of the human body, with “R” indicating right and “L” indicating left. The yellow lines represent the left and right LADI measurements (unit: mm). LADI is defined as the shortest distance from the lateral margin of the odontoid process (axis) to the medial margin of the lateral mass (atlas).

Given the high frequency of cervical motor tics in children with TD, the neck is prone to injury. Thus, the key clinical question is not merely the presence of asymmetry, but whether a greater magnitude of this asymmetry is associated with the manifestation of tic symptoms in children predisposed to TD., while a baseline level of asymmetry might be physiological, we hypothesized that in children with TD, the presence and severity of cervical tics might be related to a greater degree of radiographic asymmetry. This study aimed to investigate VBLADI value in this population, specifically testing the association between radiological asymmetry and symptoms in TD children who present with neck tics. Figure 3 shows the X-ray manifestation of widening of the lateral atlantodental interval.

Our multi-tiered statistical analysis reveals an association between widened VBLADI and specific tic symptoms. The most robust finding is that the association between head shaking and VBLADI >2.2 mm remained strong and statistically significant after adjusting for potential confounders and stringent correction for multiple comparisons (aOR = 12.64, FDR *q* = 0.035).

The initial univariate analysis (Chi-square tests) suggested several potential differences, which may have included bias from uncontrolled confounding factors. The subsequent multivariate logistic regression model, which adjusted for covariates such as age and sex, refined these associations and preliminarily identified head shaking, stretching/shrinking neck, and trunk movements as more independent correlates. However, when we applied FDR correction to address the fundamental statistical issue of multiple testing, only the association with head shaking survived this most stringent threshold.

The attenuation of the associations for stretching/shrinking neck and trunk movements could be explained by several factors. First, these symptoms may be more susceptible to influence by other factors (e.g., anxiety, fatigue, or concomitant musculoskeletal issues), diluting their specific link to atlantoaxial instability. For example, studies have shown that local mechanical imbalances cause sustained tension in the muscles around the atlantoaxial joint, particularly the suboccipital muscles. To alleviate fatigue, the body may engage in repetitive movements or postural adjustments as a form of self-regulation (39). It is known that the suboccipital muscles connect to the facial expression muscles via the galea aponeurotica, forming a myofascial chain (40). This anatomical connection allows tension or abnormal activity to transmit between the head, neck, and face. This explains why motor tics involving the head, face, mouth, and nose often co-occur in children with TD. Furthermore, Jiang et al. (41) researched that a correlation between scoliosis and TD and suggested that children with neck, shoulder and trunk tics were often associated with scoliosis. In the spinal functional activities, there are many complex pathophysiological phenomena, and TD is closely related to the structural abnormalities of the cervical spine and even the spine. This explains the more complex biomechanics involved in the complex neck tics and trunk tics. Second, it may be related to the limited sample size; while our study was powered to detect a large effect size as seen in head shaking, it may have been underpowered to detect smaller effects or effects more prone to measurement error.

Nevertheless, the persistence of significance for head shaking across models and corrections is striking and is supported by a high degree of biological plausibility. From a biomechanical perspective, neck tics may be associated with ligamentous injuries, primarily involving the alar ligaments and capsule ligaments. Firstly, it is well-established that head shaking is related to lateral neck flexion, involving the mid-to-lower cervical spine (C3-C7), the craniovertebral junction (CVJ), as well as the muscular and ligamentous systems (22). Previous autopsy studies have shown that the removal of the alar ligament increases the occurrence of lateral flexion of the upper cervical spine, and the removal of the C1-C2 joint capsular ligaments also increases lateral bending and axial rotation on the opposite side of the neck (42, 43). The alar ligament is the primary ligament that limits excessive lateral flexion and rotation of the atlanto-occipital joint complex (C0-C1) (10). Secondly, the capsular ligaments enclose each facet joint and contribute to stability during neck rotation (11). Among the four primary motions of the cervical spine—flexion, extension, axial rotation, and lateral flexion (44)—only the capsular ligaments of the facet (apophyseal) joints restrict all four movements (25). Weakening of capsular ligaments may lead to increased range of motion in all directions.

From a neural mechanism perspective, factors such as nerve compression, altered cerebrospinal fluid dynamics, and dysfunction of the central nervous system can continuously stimulate cervical proprioceptors, leading to abnormal conduction of nerve impulses and thereby triggering tic symptoms (45). Atlantoaxial joint instability may compress adjacent structures such as the vertebral artery, the upper cervical spinal cord, or the brainstem, affecting neural transmission in regions like the reticular formation and basal ganglia (35, 36, 46). Given that TD is associated with dysfunction in the basal ganglia-thalamo-cortical circuit, and considering that the brainstem is a key region regulating motor inhibition, such compressive effects may directly induce or exacerbate tic symptoms.

In conclusion, VBLADI may be a radiographic marker of CVJ dysfunction. When VBLADI ≥2.2 mm, children with TD are more likely to exhibit head shaking. The other symptoms that emerged in preliminary analyses (e.g., neck stretching/shrinking, trunk movements), while not surviving strict statistical correction, are characterized by large effect sizes (OR > 8) and may represent genuine signals worthy of prioritization in future validation studies with larger, prospective cohorts.

This discovery may prompt clinicians to consider that when children with TD come to see a doctor with shoulder and neck movements as the main or initial symptoms, there may be asymmetry in the lateral atlantodental interval, and targeted imaging examinations should be conducted to rule out cervical diseases. In terms of treatment, in addition to conventional psychological-behavioral interventions and neuropharmacological control of symptoms, targeted exercises for the neck and trunk skeletal muscles and joints to improve muscle ligament tension and stability may serve as a new idea for assisting in symptom relief.

Furthermore, this study conducted the first assessment of the relationship between the severity of TD and VBLADI, and found no statistically significant difference, indicating that VBLADI levels are not correlated with the complexity, interference, or frequency of tic symptoms. The causality among TD and VBLADI levels is not clear. Several interpretations could explain this result. First, VBLADI may act more as a “threshold” than a “gradient” metric. Once the asymmetry exceeds a certain critical threshold (e.g., 2.2 mm as used in our study), it may predispose an individual to tics, particularly cervical ones; however, beyond this point, the absolute degree of asymmetry may not linearly correlate with symptom intensity or frequency—an “on/off” effect rather than a “dimmer switch” effect. Second, the frequency, complexity, and interference of tics are likely primarily driven by core neuropathological mechanisms of TD, such as neurotransmitter dysregulation and dysfunction within cortico-striato-thalamo-cortical circuits. Cervical biomechanical factors may serve primarily as a peripheral trigger or exacerbating factor within this complex framework, influencing the manifestation but not the central drive of the disorder. Therefore, Future studies with larger, prospective cohorts and dynamic imaging assessments are warranted to unravel the precise nature of this relationship.

This study still has the following limitations. Firstly, the temporal sequence of events cannot be established, thereby precluding causal inference between VBLADI and tic symptoms. Secondly, the recruitment of participants who underwent imaging from a single center may introduce selection bias, limiting the generalizability of our findings. Although multivariate regression was used to adjust for known confounding variables, unmeasured or unrecorded potential confounders (e.g., lifestyle habits) may still affect the results. Furthermore, the reliance on electronic medical records for some clinical data may raise concerns regarding completeness and consistency.

Additionally, the sample size of 80 children with TD remains relatively small. There is also ongoing controversy regarding the normal reference range of VBLADI in the pediatric population, which varies from 1.39 to 3.9 mm in previous reports (30, 47, 48). Although the cutoff value of 2.2 mm used in this study—based on measurements from 495 Chinese children—lends some scientific rigor to the group classification (28), it should be interpreted with caution. Moreover, while previous studies have determined VBLADI values using dynamic CT or MR examinations due to the limitations of digital radiography (DR)—such as susceptibility to head positioning and a limited imaging field (29, 49)—this study utilized DR imaging for clinical practicality and reduced radiation exposure. Prospective, multi-center studies with larger sample sizes are warranted in the future to validate our findings and elucidate the underlying causal mechanisms.

In this study, DR imaging was chosen for this investigation due to the fact that it is less radiation-intensive for children than CT, easier to perform clinically, more economical, and easier to secure patient and family cooperation. Furthermore, the current state of DR imaging quality is acceptable. Professional imaging physicians are responsible for taking X-rays. The head position is promptly modified to guarantee that the child's head is as centered as possible in order to minimize manual error, based on the imaging quality.

Totally, our research has several advantages. This study is the first to reveal the LADI levels in the population of children with TD, exploring the clinical significance of the VBLADI measurement in children with TD. As far as we know, we are the first to conduct research on the correlation between TD and VBLADI, and we have found that shaking head is strongly associated with VBLADI >2.2 mm. An increase in VBLADI may be related to excessive tension in the neck muscles or ligaments, suggesting that it can be used as a reference indicator for cervical spine dysfunction. In clinical practice, for children with TD, in addition to conventional intervention measures, more emphasis should be placed on functional exercise and rehabilitation of the skeletal-muscular-joint system to improve muscle and ligament tension and stability, which may provide new ideas for assisting in symptom relief.

## 5 Conclusion

The strong and specific association between an increased VBLADI (>2.2 mm) and head-shaking tics suggests that craniocervical instability may be a significant biomechanical factor in the manifestation of this specific symptom in children with TD.

## Data Availability

The raw data supporting the conclusions of this article will be made available by the authors, without undue reservation.
